# Novel Allergen Discovery through Comprehensive De Novo Transcriptomic Analyses of Five Shrimp Species

**DOI:** 10.3390/ijms22010032

**Published:** 2020-12-22

**Authors:** Shaymaviswanathan Karnaneedi, Roger Huerlimann, Elecia B. Johnston, Roni Nugraha, Thimo Ruethers, Aya C. Taki, Sandip D. Kamath, Nicholas M. Wade, Dean R. Jerry, Andreas L. Lopata

**Affiliations:** 1 Molecular Allergy Research Laboratory, College of Public Health, Medical and Veterinary Sciences, James Cook University, Townsville, QLD 4811, Australia; shaymaviswanathan.karnaneedi@my.jcu.edu.au (S.K.); elecia.johnston@jcu.edu.au (E.B.J.); roni.nugraha@my.jcu.edu.au (R.N.); thimo.ruethers@my.jcu.edu.au (T.R.); aya.taki@unimelb.edu.au (A.C.T.); sandip.kamath@jcu.edu.au (S.D.K.); 2 Australian Institute of Tropical Health and Medicine, James Cook University, Townsville, QLD 4811, Australia; 3 Centre for Food and Allergy Research, Murdoch Children’s Research Institute, The Royal Children’s Hospital, 50 Flemington Road, Parkville, VIC 3052, Australia; 4 ARC Research Hub for Advanced Prawn Breeding, Townsville, QLD 4811, Australia; roger.huerlimann@jcu.edu.au (R.H.); nick.wade@csiro.au (N.M.W.); 5 Centre for Sustainable Tropical Fisheries and Aquaculture, College of Science and Engineering, James Cook University, Townsville, QLD 4811, Australia; 6 Centre for Tropical Bioinformatics and Molecular Biology, James Cook University, Townsville, QLD 4811, Australia; 7 Department of Aquatic Product Technology, Bogor Agricultural University, Bogor 16680, Indonesia; 8 CSIRO Agriculture and Food, Aquaculture Program, 306 Carmody Road, St Lucia, QLD 4067, Australia; 9 Tropical Futures Institute, James Cook University, 149 Sims Drive, Singapore 387380, Singapore

**Keywords:** allergy, prawn, tropomyosin, allergen, RNA-Seq

## Abstract

Shellfish allergy affects 2% of the world’s population and persists for life in most patients. The diagnosis of shellfish allergy, in particular shrimp, is challenging due to the similarity of allergenic proteins from other invertebrates. Despite the clinical importance of immunological cross-reactivity among shellfish species and between allergenic invertebrates such as dust mites, the underlying molecular basis is not well understood. Here we mine the complete transcriptome of five frequently consumed shrimp species to identify and compare allergens with all known allergen sources. The transcriptomes were assembled de novo, using Trinity, from raw RNA-Seq data of the whiteleg shrimp (*Litopenaeus vannamei*), black tiger shrimp (*Penaeus monodon*), banana shrimp (*Fenneropenaeus merguiensis*), king shrimp (*Melicertus latisulcatus*), and endeavour shrimp (*Metapenaeus endeavouri*). BLAST searching using the two major allergen databases, WHO/IUIS Allergen Nomenclature and AllergenOnline, successfully identified all seven known crustacean allergens. The analyses revealed up to 39 unreported allergens in the different shrimp species, including heat shock protein (HSP), alpha-tubulin, chymotrypsin, cyclophilin, beta-enolase, aldolase A, and glyceraldehyde-3-phosphate dehydrogenase (G3PD). Multiple sequence alignment (Clustal Omega) demonstrated high homology with allergens from other invertebrates including mites and cockroaches. This first transcriptomic analyses of allergens in a major food source provides a valuable resource for investigating shellfish allergens, comparing invertebrate allergens and future development of improved diagnostics for food allergy.

## 1. Introduction

Food allergy affects up to 10% of children and 10% of adults, and the prevalence is projected to rise [1,2]. Food allergy is caused through the ingestion of food that contains normally harmless proteins but trigger an adverse reaction in sensitised individuals [3,4]. The term “allergen” refers to a protein capable of inducing sensitisation and subsequent allergic immune responses through immunoglobulin E (IgE)-mediated type 1 hypersensitivity in patients [3,4,5,6,7].

Similar to peanut allergy, shellfish allergy is often lifelong, affects about 2% of the global population and is highly prevalent in the Asia-Pacific region and other countries where seafood consumption is high [8,9,10,11]. A recent epidemiological study from Vietnam revealed that the prevalence of shellfish allergy is as high as 4.2%, while up to 3% of adults in the USA are sensitised to shellfish [1,11].

Among shellfish-allergic individuals, shrimp allergy seems to be the most prominent crustacean allergy and remains to be difficult to diagnose and manage, for multiple reasons. Shrimp accounts for one of the most prevalent events of food-derived anaphylactic reactions after peanuts and tree nuts [12,13,14,15]. 

The management of shrimp allergy is often challenging due to immunological cross-reactivity to molecularly similar allergens [12,13,16,17,18,19,20,21,22]. The similarity of shrimp allergens to proteins of other shellfish species, including crabs and lobsters, and other invertebrates such as house dust mites (HDM) and cockroaches, can induce unexpected allergic reactions [15,16,17,18,19,20,23,24]. Although this cross-reactivity has been observed in the clinical setting, the underlying molecular basis is not well understood [25,26]. 

Over the past decades, more than 2000 allergens have been characterised and are accessible via online databases, including the World Health Organization & International Union of Immunological Societies (WHO/IUIS) Allergen Nomenclature database (www.allergen.org), which has the most stringent inclusion criteria, and the highly peer-reviewed AllergenOnline: The Food Allergy Research and Resource Program (FARRP) Allergen Protein database (www.allergenonline.org) [7,27,28,29]. Allergen discovery is traditionally conducted using whole protein extracts and identification of IgE antibody binding proteins [4,30,31,32,33]. However, this approach has many limitations, including low sensitivity of the technology and small patient cohorts that does not allow the detection of all allergenic proteins [34].

**Figure 1:**
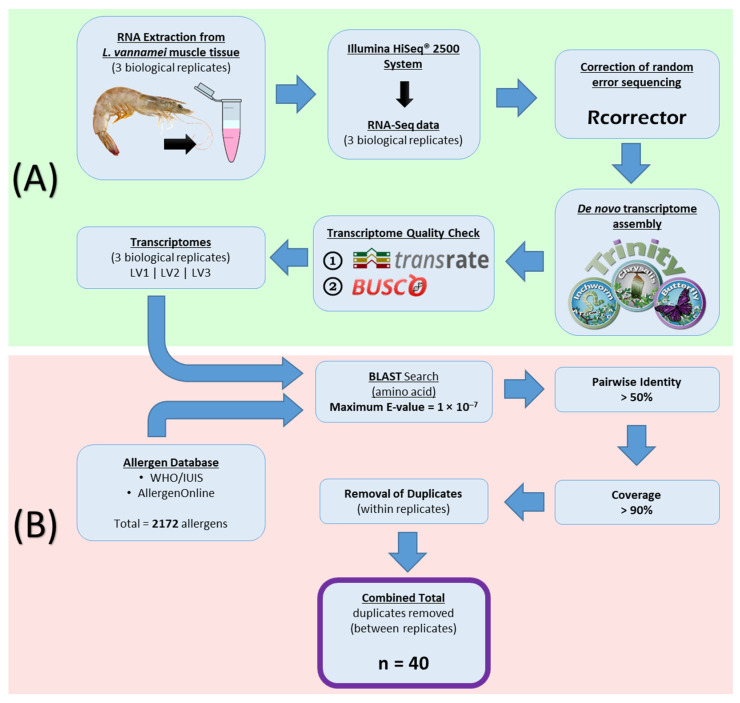
Schematic representation of (**A**) de novo transcriptome assembly and (**B**) transcriptomic analysis used in the identification of allergens in shrimps. The example shown here is for *L. vannamei*. LV1, LV2, and LV3 represent the three biological replicates of *L. vannamei* samples. ‘n’ equals the number of allergens identified in *L. vannamei*. A total of 40 allergens were identified.

Here we report the first complete transcriptomic analysis of a food allergen sources, with a focus on shrimp (shellfish) allergens. The transcriptomes of five frequently consumed shrimp species were assembled de novo and analysed for the presence of similar amino acid (AA) sequences to 2172 allergens in the WHO/IUIS Allergen Nomenclature and AllergenOnline databases (Figure 1). This analysis utilises an AA sequence similarity or pairwise identity cut-off value of 50%, derived from past studies that observed that high AA sequence identity (>50%) between homologous proteins/allergens is the most predictive comparison to determine whether the allergens are cross-reactive or whether the protein is likely to be an allergen [28,35]. The current study aims to predict the latter; the likelihood of a shrimp protein to be an allergen.

## 2. Results

### 2.1. Assessment of 15 Assembled Transcriptomes

The completeness and assembly quality of the transcriptomes were analysed using different assessment tools. RNA sequencing, using Illumina HiSeq^®^ 2500 (Illumina Australia and New Zealand, VIC, Australia), produced 125 bp (base pairs) paired-end sequencing data with a total number of paired-end reads for each sample of approximately 20 million reads. The de novo assembly of 15 samples (three replicates each for five shrimp species) resulted in 28,101 to 42,510 contigs (Table 1). All 15 samples had more than 87% of read pairs that mapped back to the contigs within the assembled transcriptome. TransRate scores (assembly scores) for each of the 15 transcriptomes were approximately 0.4 (Table 1). BUSCO (Benchmarking Universal Single-Copy Orthologs) results, overall, had a complete genes (C) score ranging 43–67%, fragmented genes (F) score ranging 16–26%, and missing genes (M) score in the range of 14–32% (Table 1). The transcriptomes of *P. monodon* and *F. merguiensis* had the highest values for completeness (BUSCO C score) (Table 1). These two shrimp species also had the highest number of contigs and assembly size.

### 2.2. Known and Potential Allergens Identified within the Shrimp Transcriptomes

**Figure 2:**
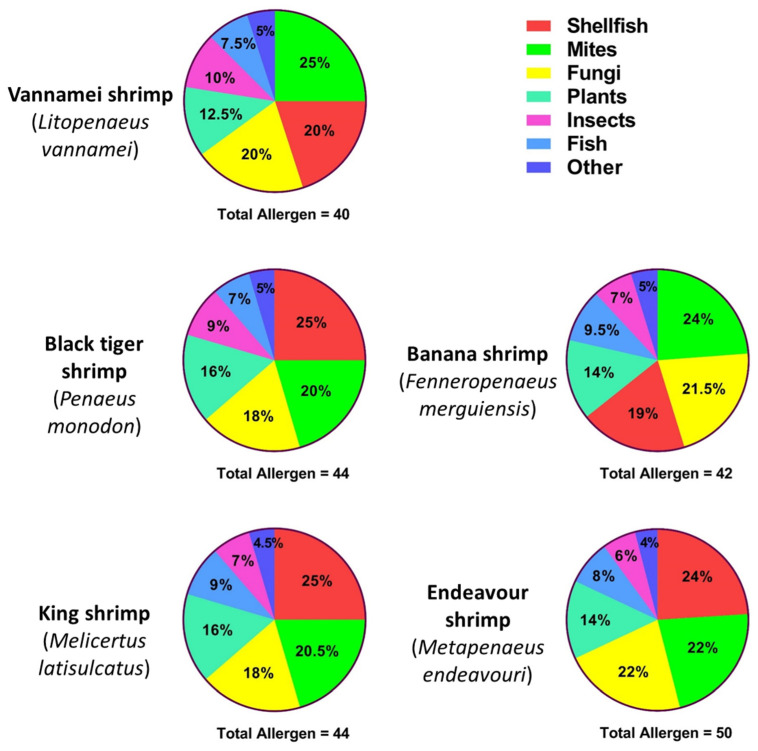
Total allergens identified from the transcriptomic analysis in each of the five shrimp species, distributed based on the matched allergen’s source. The distribution amongst different groups of allergen sources is shown in percentages and arranged in descending order.

BLAST analysis identified large numbers of allergen sequence matches, primarily with allergens known in shellfish, mites and fungi. After duplicate removal, the results yielded 40 allergen sequences identified in whiteleg shrimp (*L. vannamei*), 44 in black tiger shrimp (*P. monodon*), 42 in banana shrimp (*F. merguiensis*), 44 in king shrimp (*M. latisulcatus*) and 50 in endeavour shrimp (*M. endeavouri*) (Figure 2). Approximately two-thirds of allergen AA sequences that matched with all five shrimp species transcriptomes belonged to shellfish, mites and fungi species (Figure 2). The remaining allergen AA sequences belonged to plants, insects, fish and other allergen sources.

### 2.3. Identification of Known Crustacean Allergens

**Figure 3:**
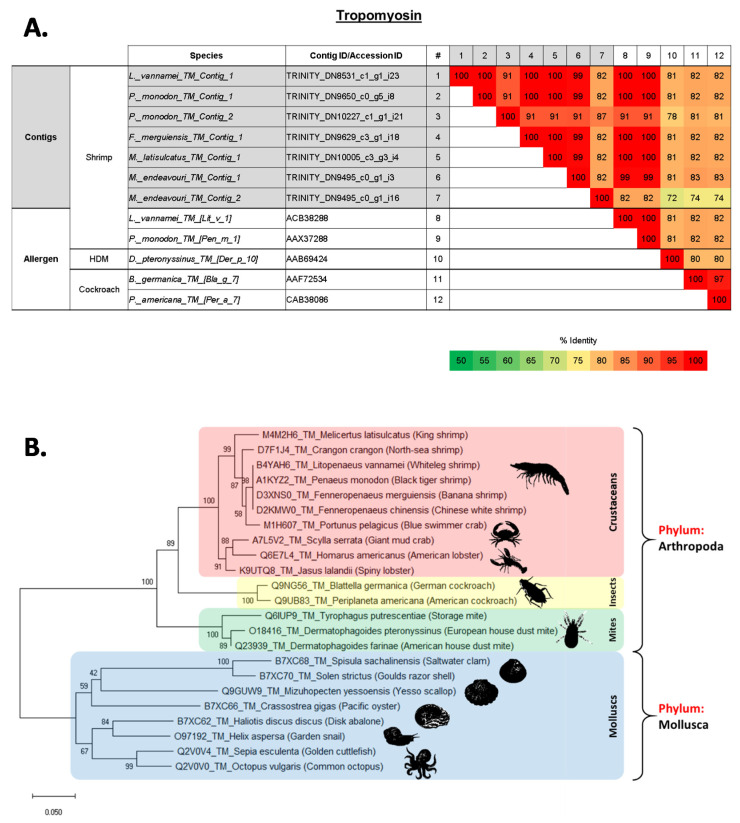
(**A**) Comparison of amino acid sequence identities of (1–7) contigs from five shrimp species that matched with tropomyosin (TM) allergens, (8–9) known shrimp TM allergens and (10–12) house dust mite and cockroach TM allergens. The sequence identities were calculated using multiple sequence alignment in Clustal Omega (EMBL-EBI). (**B**) Molecular phylogenetic tree based on published amino acid sequences of Tropomyosin (TM) from edible crustacean and mollusc species, and allergy causing mite and insect species. The branches consist of UniProt ID/Genbank Accession ID, species name, followed by common name in brackets. The numbers next to the branches indicate the bootstrap test percentage of 10,000 replicate trees.

In total, seven crustacean allergens were identified among the five shrimp species, and some species seem to have up to two isoforms of an allergen. Contigs that matched with the major shrimp allergen tropomyosin (TM) were identified in all five species, with some species having more than one contig representing this allergen (Figure 3A and Figure S1). TM_Contig_1 from *L. vannamei* has a 100% AA sequence identity with the previously recorded and IUIS registered Lit v 1 (ACB38288). This is a similar finding to TM_Contig_1 from *P. monodon*, which has a 100% sequence identity with Pen m 1 (AAX37288). Both contigs also match with a 100% similarity with each other (Figure 3A). Overall, TM_Contig_1 from all five species showed a high sequence similarity, pairwise identity (PI) of 99–100%, with Lit v 1 and Pen m 1. However, TM_Contig_2 of *P. monodon* and *M. endeavouri* only showed a pairwise identity (PI) of 91% and 82%, respectively, with Lit v 1/Pen m 1. The inclusion of HDM and cockroach tropomyosin allergens, Der p 10 (AAB69424), Bla g 7 (AAF72534) and Per a 7 (CAB38086), in the analyses demonstrated that all three allergens have more than 70% PI with shrimp TM, ranging from 72% to 83%. The analysis of molecular phylogenetic trees using known AA sequences of TM, revealed that TM between crustaceans is very similar to each other, and also to insect and mite TMs (Figure 3B). In contrast, TM AA sequences of molluscs, which are in the context of allergy diagnosis often grouped as “shellfish” together with crustaceans, seem to be distantly related. 

**Figure 4:**
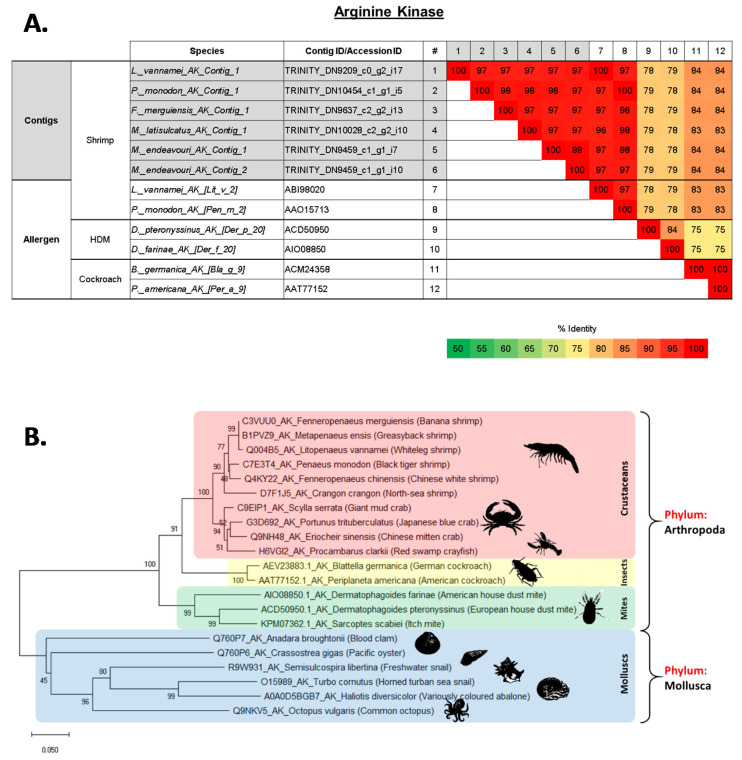
(**A**) Comparison of amino acid sequence identities of (1–6) contigs from five shrimp species that matched with arginine kinase (AK) allergens, (7–8) known shrimp AK allergens and (9–12) house dust mite and cockroach AK allergens. The sequence identities were calculated using multiple sequence alignment in Clustal Omega (EMBL-EBI). (**B**) Molecular phylogenetic tree based on published amino acid sequences of Arginine kinase (AK) from edible crustacean and mollusc species, and allergy causing mite and insect species. The branches consist of UniProt ID/Genbank Accession ID, species name, followed by common name in brackets. The numbers next to the branches indicate the bootstrap test percentage of 10,000 replicate trees.

The allergen arginine kinase (AK) was identified in all five shrimp species, while *M. endeavouri* had two contigs for this allergen. All six contigs were highly similar to each other and to the published AK allergens in *L. vannamei*, Lit v 2 (ABI98020), and *P. monodon*, Pen m 2 (AAO15713), with more than 95% PI (Figure 4A and Figure S2). These sequences are also similar to the published cockroach AK allergens in *Blattella germanica*, Bla g 9 (ACM24358), and *Periplaneta americana*, Per a 9 (AAT77152), (83–84% identity). In contrast, these were different to the published HDM AK allergens in *Dermatophagoides pteronyssinus*, Der p 20 (ACD50950) and *Dermatophagoides farinae*, Der f 20 (AIO08850) (78–79% identity) (Figure 4A). Similar to TM, published AA sequences of crustacean AK are more closely related to each other as well as to insects and mites, but not closely related to molluscs (Figure 4B).

**Figure 5:**
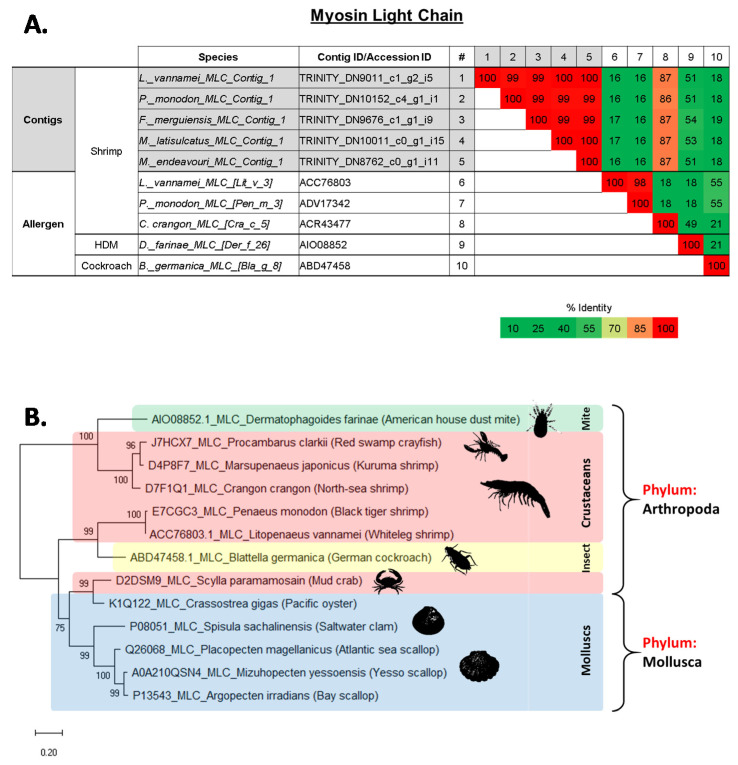
(**A**) Comparison of amino acid sequence identities of (1–5) contigs from five shrimp species that matched with myosin light chain (MLC) allergens, (6–8) known shrimp MLC allergens and (9–10) house dust mite and cockroach MLC allergens. The sequence identities were calculated using multiple sequence alignment in Clustal Omega (EMBL-EBI). (**B**) Molecular phylogenetic tree based on published amino acid sequences of Myosin light chain (MLC) from edible crustacean and mollusc species, and allergy causing mite and insect species. The branches consist of UniProt ID/Genbank Accession ID, species name, followed by common name in brackets. The numbers next to the branches indicate the bootstrap test percentage of 10,000 replicate trees.

The allergen myosin light chain (MLC) was identified in all five shrimp species, with one contig each, which demonstrated almost identical AA sequences to each other. Interestingly, they were different to the published MLC allergens from *L. vannamei*, Lit v 3 (ACC76803), or *P. monodon*, Pen m 3 (ADV17342), with only 16–17% PI (Figure 5A and Figure S3). Instead, these sequences are more similar to the *Crangon crangon* (North-sea shrimp) MLC allergen, Cra c 5 (ACR43477) with 86–87% PI (Figure 5A). The contigs were more closely related to the American HDM, *D. farinae*, MLC allergen, Der f 26, (51–54% identity) than the German cockroach, *B. germanica*, MLC allergen, Bla g 8 (18–19% identity) (Figure 5A). Molecular phylogenetic tree analyses on the distance of MLC among edible crustaceans, molluscs and allergy-causing mites confirmed that not all crustacean MLC are closely related to each other. Overall, some MLC proteins seem to be similar to molluscs. For example, mud crab (*S. paramamosain*) is more closely related to mollusc MLC than shrimp and crayfish (Figure 5B). Black tiger shrimp (*P. monodon*) and whiteleg shrimp (*L. vannamei*) contain MLC that are distantly related to kuruma shrimp (*M. japonicus*) and north-sea shrimp (*C. crangon*), however closely related to MLC from mollusc and German cockroach (*B. germanica*) (Figure 5B). 

**Figure 6:**
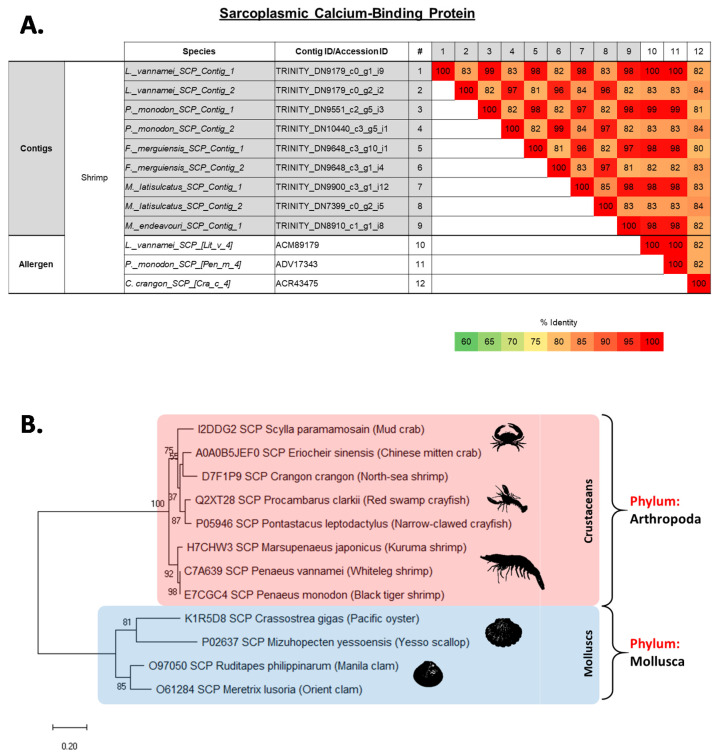
(**A**) Comparison of amino acid sequence identities of (1–9) contigs from five shrimp species that matched with sarcoplasmic calcium-binding protein (SCP) allergens and (10–12) known shrimp SCP allergens. The sequence identities were calculated using multiple sequence alignment in Clustal Omega (EMBL-EBI). (**B**) Molecular phylogenetic tree based on published amino acid sequences of Sarcoplasmic calcium-binding protein (SCP) from edible crustacean and mollusc species, and allergy causing mite and insect species. The branches consist of UniProt ID/Genbank Accession ID, species name, followed by common name in brackets. The numbers next to the branches indicate the bootstrap test percentage of 10,000 replicate trees.

The allergen sarcoplasmic calcium-binding protein (SCP) was identified with two contigs in four of the analysed shrimp species, while *M. endeavouri* had only one contig. SCP_Contig_1 from all five shrimp species were highly similar to each other and also with the published SCP allergen in *L. vannamei*, Lit v 4 (ACM89179) and *P. monodon*, Pen m 4 (ADV17343) with PI close to 100%, and 80–83% with the published SCP allergen in *C. crangon*, Cra c 4 (ACR43475) (Figure 6A and Figure S4). In contrast, SCP_Contig_2 from the four species, except *M. endeavouri*, were 82–84% identical to Lit v 4, Pen m 4 and Cra c 4, with the last one having a slightly higher match than the first two (Figure 6A). Unlike MLC, but similar to TM and AK, the published AA sequences of SCP in a phylogenetic tree analysis portrayed that all SCP from edible crustaceans and molluscs are very closely related to other species within the same phylum, but distantly related between the phyla (Figure 6B). No allergen sequences were available for comparison from insects or mites.

Seven contigs matched with Troponin C (TNC) across the five shrimp species, with *M. latisulcatus* and *M. endeavouri* having two contigs each whilst the other three shrimp species having one each. All seven contigs were moderate to highly similar to each other and with the published TNC allergens in *P. monodon*, Pen m 6 (ADV17344) and *C. crangon*, Cra c 6 (ACR43478), with PI at a range of 81–100% (Figures S5 and S6). The PI of shrimp TNC with cockroach and storage mite TNC allergens were at a range of 57–65% (Figure S5). Meanwhile, only one contig from each shrimp species matched with Troponin I (TNI), and they were all highly identical to each other (PI: 87–99%) but were only moderately identical to the published TNI allergen in the narrow-clawed crayfish *P. leptodactylus*, Pon l 7 (P05547) (PI: 78–88%) (Figures S7 and S8). Similarly, only one contig matched with Triosephosphate isomerase (TIM) in each shrimp species and were all highly identical to each other and also with the published TIM allergen in *C. crangon*, Cra c 8 (ACR43476), (PI: 87–99%) (Figures S9 and S10). However, they had lower PI to American HDM TIM allergens, Der f 25.01 (AGC56216) and Der f 25.02 (AIO08860), with PI values at a range of 66–69% (Figure S9).

### 2.4. Abundance of Known Crustacean Allergen Transcripts Varies Between Shrimp Species

**Figure 7:**
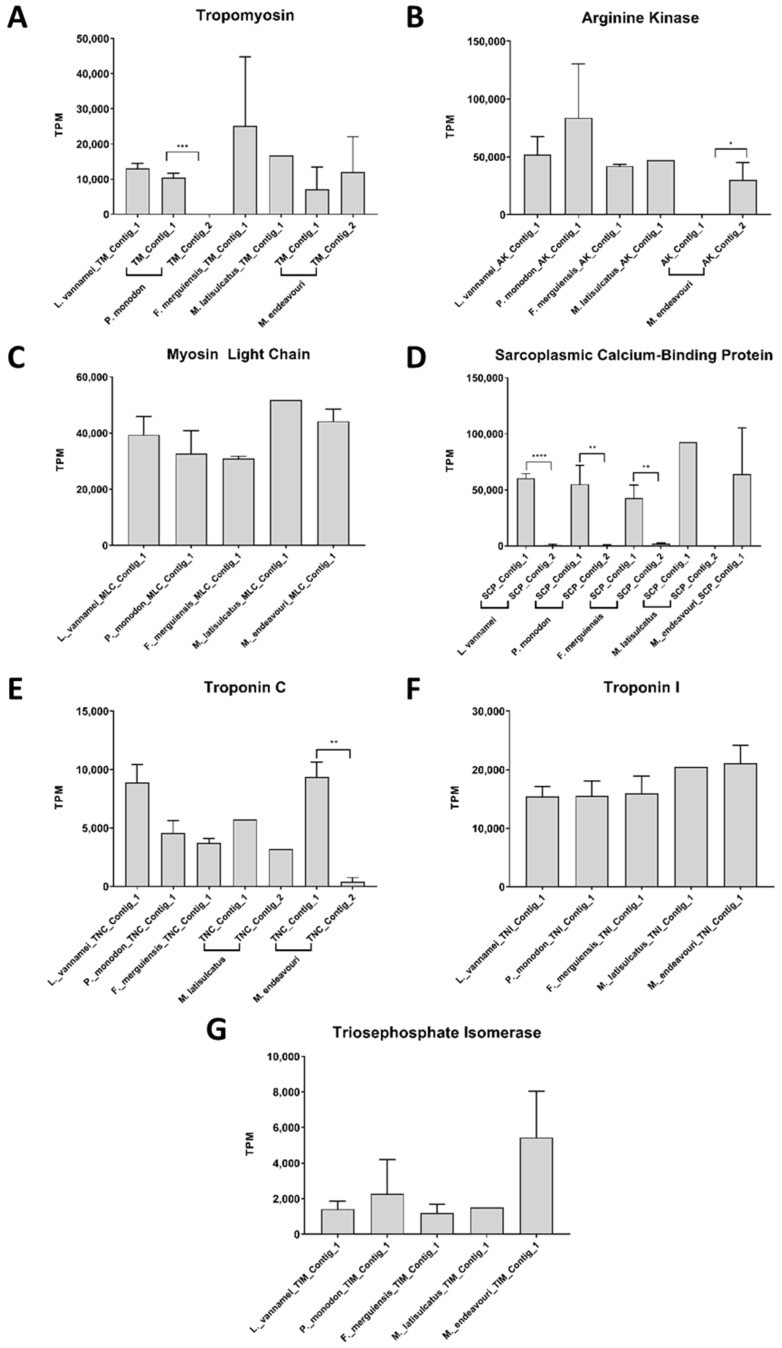
Abundance estimation values in transcript-per-million (TPM) for contigs in the 5 analysed shrimp species that matched with shrimp allergens. (**A**): tropomyosin, (**B**): arginine kinase, (**C**): myosin light chain, (**D**): sarcoplasmic calcium-binding protein, (**E**): troponin C, (**F**): troponin I, and (**G**): triosephophate isomerase. *t*-tests were employed to measure the significance of difference between two contigs from the same species, if present (*: *p* ≤ 0.05, **: *p* ≤ 0.01, ***: *p* ≤ 0.001, ****: *p* ≤ 0.0001).

The average expression or mean abundance, measured in transcripts-per-million (TPM), of TM across all five species are at a range 10,000–15,000 TPM (Figure 7A). Comparing the difference in abundance between the two TM contigs within the same species (*P. monodon* and *M. endeavouri*), TM_Contig_2 of *P. monodon* was significantly lower than TM_Contig_1 (Figure 7A). Meanwhile, there was no significant difference between TM_Contig_1 and TM_Contig_2 of *M. endeavouri* (Figure 7A). The mean abundance for AK was approximately 40,000–80,000 TPM in all five species (Figure 7B). Comparing the abundance of the two AK contigs in *M. endeavouri*, AK_Contig_1 was significantly lower than AK_Contig_2 (Figure 7B). The mean abundance of MLC was approximately 30,000–50,000 TPM in all species (Figure 7C). Meanwhile, for SCP, the mean abundance was between 40,000 and 90,000 TPM in all species (Figure 7D). Interestingly, SCP_Contig_1 of *L. vannamei, P. monodon,* and *F. merguiensis* were all significantly higher than their respective SCP_Contig_2 (Figure 7D). The same pattern is also observed for *M. latisulcatus*, however the significance could not be statistically confirmed. In contrast, for *M. endeavouri,* TNC_Contig_1 was significantly higher than TNC_Contig_2 (Figure 7E). Overall, the mean abundance value for TNC was 4000–10,000 TPM for all five shrimp species (Figure 7E). As for TNI and TIM, the mean abundance values for all five shrimp species were approximately 16,000–20,000 TPM (Figure 7F) and 2000–6000 TPM, respectively (Figure 7G).

**Figure 8:**
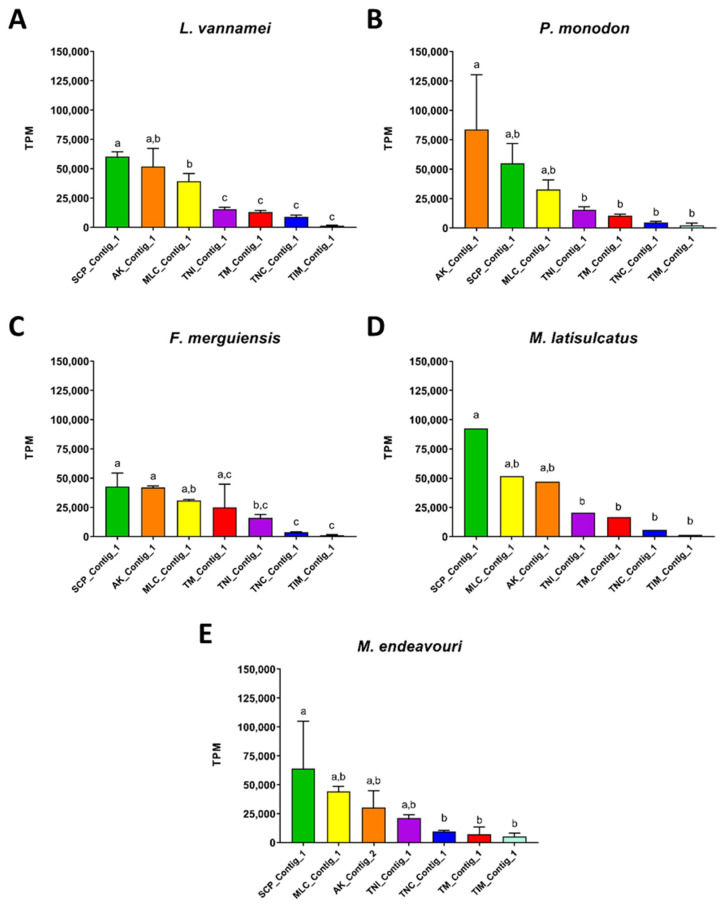
Abundance estimation in transcript-per-million (TPM) for contigs that matched with shrimp allergens in the 5 analysed shrimp species. (**A**): *L. vannamei*, (**B**): *P. monodon*, (**C**): *F. merguiensis*, (**D**): *M. latisulcatus*, (**E**): *M. endeavouri.* ANOVA tests were employed to measure the significance of difference between the seven shrimp allergens. Only one contig with the highest Pairwise Identity with known shrimp allergens was included where there was more than one contig for one allergen in each species. The contigs are arranged in descending order of on their abundance. Allergen abundance with the same letter are not significantly different to each other.

Subsequently, the differences in abundance of each allergen within individual shrimp species was examined. When there was more than one contig for an allergen, only the contig with the highest PI was used for the analysis. In all species, the top three highest expressed allergen transcripts were SCP, AK and MLC (Figure 8). In fact, SCP was the highest expressed allergen gene in all species except *P. monodon*, where AK was higher (Figure 8B). In descending order of abundance, these three allergens are followed by TNI, TM, TNC and TIM (Figure 8). However, in *F. merguiensis*, TM was higher than TNI, TNC and TIM (Figure 8C). Additionally, in *F. merguiensis,* the abundance of TM was not significantly different to the three highly abundant allergens, SCP, AK and MLC (Figure 8C).

### 2.5. Evolutionary Relationship of Shellfish Allergens TM, AK, MLC and SCP

The evolutionary distance of shrimp TM, AK, MLC and SCP were analysed among other edible crustacean and mollusc species, as well as allergy causing mite and insect species. The generated molecular phylogenies of all four shrimp proteins showed close affinities to homologues of other crustaceans such as crab, lobster and crayfish (see Figure 3, Figure 4, Figure 5 and Figure 6). However, homologues of mollusc, grouped often with crustacean as “shellfish”, have a distant relationship to crustacean allergens. Molecular phylogenetic analyses of TM and AK demonstrated that allergy-inducing mite and insect homologues are closer in relation to TM and AK from shrimp compared to molluscs. This observation is supported by a recent study by Nugraha et al. where IgE antibody binding epitopes demonstrated shared protein regions of clinical importance [36]. MLC of German cockroach is found to have a closer molecular relationship to the black tiger shrimp and whiteleg shrimp, whilst the MLC of the American house dust mite is closely related to MLC of a different subset of crustaceans, including the north-sea shrimp, kuruma shrimp, and red swamp crayfish. Another important finding is that the crustacean MLC of mud crab seems to have a closer relationship with homologues from the mollusca phylum, especially the pacific oyster, but not to other crustaceans. Molecular phylogenetic analysis of SCP shows a defined distance between the crustacean and mollusc SCP. No insect or mite SCP could be included in these analyses as there are no AA sequence data available for insect or mite SCP on NCBI Genbank or UniProt databases.

### 2.6. Discovery of Unreported Allergens in Shrimp

In addition to the previously identified shellfish allergens that were confirmed in the five shrimp transcriptome analyses above, up to 39 non-shellfish allergens matched with the shrimp transcriptomes. Among these newly discovered potential allergens, some are highly likely candidates to be potential novel allergens in shrimps due to their high % PI (>70%) to other known allergens. While this study searched against all known allergens, the main focus was on allergens from sources related to shrimps such as invertebrates and fish. Other known allergens that matched with the shrimp transcriptomes such as glyceraldehyde-3-phosphate dehydrogenase (G3PD) and cyclophilin, from plant (wheat) and fungi sources, were not included in Table 2 due to a lack of current evidence of homologous allergens occurring between these phylogenetically distant species. Even though fish are phylogenetically distant from shrimps, matched fish allergens are included in Table 2 due to reports of potential cross-reactivity amongst seafood (fish and shrimp) allergens. Therefore, the refined list of highly likely potential allergens includes heat shock protein 70 (HSP70), alpha-tubulin, chymotrypsin, beta-enolase, and aldolase A (Table 2).

The other two mite allergen AA sequences that matched with a PI of more than 70% to the shrimp transcriptomes are alpha-tubulin (Der f 33, AIO08861) and chymotrypsin (Der f 6, AAP35065) of the American HDM *D. farinae* (Table 2). Mite alpha-tubulin had a PI of 81% with contigs/transcripts of all five shrimps. Meanwhile, mite chymotrypsin had a slightly lower PI (78–80%) and matched with all five shrimp species (Table 2).

There was an additional allergen AA sequence with high similarity (>70%) in all five shrimp species: Beta-enolase (Sal s 2, ACH70932) of the Atlantic salmon *Salmo salar*. Fish allergen beta-enolase was highly similar to the transcripts across all five shrimp species with a PI of approximately 74% (Table 2). Another fish allergen was highly similar to contigs/transcripts in the analysed shrimps: Aldolase A, also known as fructose bisphosphate aldolase A, is a known allergen of the yellowfin tuna *Thunnus albacares* (Thu a 3, CAX62602). This allergen matched with a PI of 70.1% with contigs from both the banana and endeavour shrimps, but with a lower PI match with contigs from whiteleg (66%), black tiger (64.9%) and king (69.6%) shrimps (Table 2).

## 3. Discussion

Previous allergen discovery studies applying traditional protein isolation and immunological assay methods, characterised seven allergenic proteins in shellfish, including the major allergen TM, in addition to AK, MLC, SCP, TNC, TNI and TIM [15,27]. All seven allergens, except TNI, were identified in various shrimp species [27]. However, increasing clinical reports of allergic reactions to various species within the crustacean and mollusc groups, as well as allergic cross-reactivity of shrimp-allergic patients to other allergen sources, demands a full analysis of all potential allergenic proteins [15,37,38]. The current study utilised an advanced transcriptomic approach to discover and compare the whole repertoire of shrimp allergens in addition to putative novel allergens. This approach generated transcriptomes from five shrimp species and subsequent BLAST analyses against all known allergen AA sequences identified up to 50 allergens. Most of the identified allergens belong to the groups of shellfish (19–25%) and mite (20–25%) allergens. It is important to note that shellfish, in the context of seafood consumption and not based on phylogenetic relationships, consists of crustaceans (shrimp) and molluscs, which are often combined when analysing related allergens in the context of patient diagnosis [15,36,39]. 

The major shellfish allergen TM was identified in all five shrimps, and for the first time here reported in banana and endeavour shrimp [40]. The transcript abundance varied considerably between the species however, statistical analysis demonstrated no statistically significant differences. Interestingly, the AA sequences of four species was 100% identical, except for endeavour shrimp, which differed by 1%. The demonstrated 100% identity between the TM from whiteleg (Lit v 1) and black tiger shrimp (Pen m 1) was previously reported and, validates the in silico approach used in this study [15]. Furthermore, we demonstrated for the first time that TM from banana and king shrimp also exhibited 100% identity to Lit v 1 and Pen m 1. In contrast, the previously reported AA sequence of TM allergen from king shrimp (Mel l 1; AGF86397) shares 95% AA identity with Pen m 1 [41]. Importantly, this difference has been linked to species-specific allergenicity in patients and needs to be followed up in subsequent clinical studies [41]. The additional TM contig (Contig_2) in black tiger and endeavour shrimps are potentially isoallergens. The IUIS Allergen Nomenclature identifies an isoallergen to be two proteins from the same species, or family of species, with the same biological function and with similar biochemical properties including more than 67% AA sequence identity and similar molecular size [7]. This high AA sequence identity (PI: >70%) of the house dust mite (HDM) and cockroach TM allergens (Der p 10, Bla g 7 and Per a 7, respectively) with all the analysed shrimp TMs indicate a likelihood of all these invertebrate allergens of being immunologically cross-reactive. As previously established, an AA sequence identity of more than 70% would demonstrate a highly likely possibility of cross-reactive IgE antibody binding to these allergens [28,34,35]. Clinical studies have previously demonstrated a phenomenon named ‘HDM-cockroach-shrimp’ cross-reactivity, and here we provide conclusive molecular data on the AA sequence similarity of a major shrimp allergen with other invertebrate species [23,42,43,44,45,46]. 

AK, an important enzymatic protein that regulates the cellular ATP levels of invertebrates, is a heat labile protein (38–45 kDa) and highly concentrated in muscle tissue [47]. All five analysed shrimp species demonstrated very high AA sequence similarity with each other (97–100%), indicating that shrimp allergic patients sensitised to AK would most likely react to all five shrimp species. AK is also considered an important allergen amongst insects and mites and potentially a pan-allergen implicated in cross-reactivity between invertebrate species [48,49,50]. All five shrimp AKs identified in this study are highly likely allergens with high AA sequence identities (>70%) to AKs from mites (Der p 20; Der f 20) and cockroaches (Bla g 9; Per a 9). Furthermore, the two AK contigs (PI: 99%) found in endeavour shrimp indicate that they are potential variants of isoallergens due to their PI being over 90% [7]. 

MLC is part of a large macromolecular complex in muscle tissue consisting of two heavy and four light chains. Two different MLC proteins were previously reported as allergens in crustaceans, the essential MLC1 (~18 kDa) and the regulatory MLC2 (~20 kDa) [51,52]. Due to their very low AA sequence PI (<20%), MLC1 and MLC2 are not considered isoforms but two different proteins [53]. Shrimp MLC2 allergens have been identified in the whiteleg shrimp (Lit v 3) and black tiger shrimp (Pen m 3), whilst shrimp MLC1 was reported in North-sea shrimp (Cra c 5) and brine shrimp (Art fr 5) [30,54,55]. Measuring the PI of the five potential MLC allergen AA sequences identified in each species, we demonstrate that all five MLCs are very likely to be MLC1. We established in our study for the first time that this potential allergen is present in all five analysed shrimp species, in addition to the previously reported MLC2 allergen in the whiteleg shrimp and black tiger shrimp. Furthermore, this study also suggests that MLC from HDM (Der f 26) and cockroach (Bla g 8) are most likely MLC1 and MLC2, respectively, explaining the close molecular phylogenetic relationship to crustacean, but not to molluscs.

Another crustacean allergen involved in invertebrate muscle contraction is SCP (20–24 kDa), through binding of calcium ions [56,57]. We identified two different SCP contigs for each shrimp species (except endeavour shrimp), with PIs at 81–85%, implicating the presence of SCP isoallergens. However, the significantly low abundance of SCP_Contig_2 concludes that the AA sequence of SCP_Contig_1 holds a greater relevance even though both contigs will contribute to the overall amount of SCP present in shrimps. Other muscle regulatory proteins identified include the protein troponin. Troponin is composed of three subunits, suffixed C, I and T, with Troponin C and I being registered as allergens. TNC has been identified as an allergen in various crustaceans, including black tiger shrimp (Pen m 6), cockroaches and the storage mite [55,58,59,60,61]. Our study demonstrated for the first time this putative allergen in whiteleg, banana, king and endeavour shrimp and TNI in all five analysed shrimp species. TIM, an enzyme that is involved in glucose metabolism, is a registered allergen in north-sea shrimp, red swamp crayfish, American HDM, octopus and wheat [55,62,63,64,65]. TIM is now identified in all five shrimp species analysed in the current study. TNC, TNI and TIM seem to be highly conserved among shrimp species with sequence homology higher than 80%, 78% and 87%, respectively.

The correlation between transcriptomic (mRNA) and proteomic abundance of proteins has been recognised in past studies using well-established murine models [66]. This was, however, only a moderate positive correlation of *R*^2^ = 0.41, as protein abundance may also be influenced by other factors such as the rate of protein production and turnover, translation efficiency and possible habitat adaptations that take place before and during translation. Therefore, measuring mRNA or transcriptomic abundance may not provide an accurate measurement of protein abundance within an organism. Nevertheless, a study on European HDM allergen mRNA abundance concluded that allergens have a higher abundance than non-allergens, and their results were similar to homologous allergens identified in American HDM from a different study [67,68]. 

In our study, we aimed to investigate the abundance of all known crustacean allergens in terms of mRNA abundance. By comparing registered crustacean allergens within each shrimp species, the mRNA transcripts for SCP, AK and MLC were shown to be the most abundant in all five species. While TM is considered the major shrimp allergen, the abundance of transcripts was, in comparison to the other allergens, significantly lower. However, the protein abundance of TM is known to be very high [31,32] as it is a major component of muscle fibres (regulator of actin filaments). The muscle system in shrimp is very extensive, totalling more than half the total body weight. Therefore, future studies will measure the protein abundance using mass-spectrometry of known and potential shrimp allergens identified in this study and compare the mRNA and protein abundance of these allergens.

In addition to identifying and comparing known crustacean allergens between five different shrimp species, this study also aimed to identify the complete repertoire of potential shrimp allergens. This study successfully identified, in addition to the seven known crustacean allergens, up to 39 potential novel shrimp allergens, registered in other allergen sources. Three of these shrimp proteins, HSP70, alpha-tubulin and chymotrypsin have very high matches to known mite allergens for all five analysed shrimp species. These three proteins are registered allergens in different mite and insect species [27,69,70,71,72]. Clinical studies frequently report cross-allergic reactions of patients to crustacean as well as mites and insects, named the “crustacean-mite-insect syndrome” [20,42,44,46,73,74,75]. We report here the most likely allergens forming the underlying molecular basis for this not well-understood clinical phenomenon. Furthermore, the current study, for the first time, identified allergens that are possibly responsible for clinical cross-reactivity between shrimp and fish [25]. Beta-enolase and aldolase A, enzymatic proteins of the glycolytic pathway, were identified as heat labile allergens in various fish species and chicken [15,76,77]. Our findings implicate the possible importance of both proteins as strong candidate allergens in shrimps. In addition, other proteins that were identified to be potential allergens include cyclophilin and G3PD. These two allergens were not included in the main results of this study as they were identified from plant and fungi species (*Triticum aestivum* and *Aspergillus fumigatus*) that are phylogenetically distant to shrimps. However, there is emerging evidence of patients suffering from shrimp allergy also being sensitised to plant and fungi allergens [25,26]. The allergen cyclophilin is generally found in dust mite, fungi and plants, and demonstrates strong IgE-binding [63,78]. Meanwhile, G3PD, an enzymatic protein of the glycolysis similar to aldolase A and beta-enolase, has been identified as allergens recently in cockroach and fish [27,79].

In conclusion, this study accomplished the comparative analyses of all known shrimp allergens derived from the transcriptomes of five different shrimp species, assembled de novo from raw RNA-Seq data. The identification of previously characterised shrimp allergens validated the comprehensive allergen identification approach utilised in this study. The difference in transcriptomic abundance of different allergens across shrimp species may have clinical and diagnostic significance. Importantly, up to 39 additional shrimp transcripts that matched with allergenic proteins in mite, insects, fish, fungi and plants were identified. These include shrimp proteins that are highly likely to be potential allergens such as HSP70, alpha-tubulin, chymotrypsin, beta-enolase and aldolase A, however these are yet to be identified as true shrimp allergens. Future studies will focus on the protein abundance of known and potential allergens from shrimp and examine the IgE antibody-binding capacity of purified forms of these proteins to confirm clinical sensitisation in patients with shellfish allergy, and consequently, determine if these potential allergens are true shrimp allergens. 

## 4. Materials and Methods

### 4.1. Sample Selection

Specimen of the five species of shrimps (*Litopenaeus vannamei*, *Penaeus monodon Fenneropenaeus merguiensis, Melicertus latisulcatus* and *Metapenaeus endeavouri*) were supplied by the Commonwealth Scientific and Industrial Research Organisation (CSIRO) based in Queensland, Australia. *L. vannamei* and *P. monodon* samples originated from aquaculture farms whilst the other three species were caught as part of the CSIRO Northern Prawn Fishery Surveys from the benthic trawls in the Gulf of Carpenteria, Australia [80]. The shrimps were immersed in an ice-seawater slurry for a few minutes immediately after being caught, to be euthanised. Species-specific reference material was utilised to identify the species of shrimps [81]. Muscle tissue was then removed and stored in RNAlater^TM^ (Invitrogen, Carlsbad, CA, USA) [82]. *P. monodon* samples were collected as described by Huerlimann et al. (2018) [83]. Total RNA was extracted from the muscle tissue of three randomly selected adult shrimps of each of the five shrimp species (total of 15 samples) with an RNeasy Universal Extraction kit (QIAGEN, Hilden, Germany) using manufacturer’s instruction in an RNase-free laboratory [82]. RNA concentration, quality and purity were assessed using a Nanodrop UV spectrophotometer (Thermo Fisher Scientific, Wilmington, DE, USA) and Agilent Bioanalyzer (Agilent Technologies, Santa Clara, CA, USA), before being selected for sequencing. 

### 4.2. Illumina Library Preparation and RNA Sequencing

All 15 samples were sequenced via Illumina HiSeq^®^ 2500 System (Illumina Australia and New Zealand, VIC, Australia). Before sequencing, samples were quality checked with the Bioanalyzer RNA 6000 nano reagent kit (Agilent); and Illumina libraries were prepared using the TruSeq Stranded mRNA Library Preparation Kit (Illumina) according to established protocols at the Australian Genome Research Facility (AGRF). The resulting libraries were checked again with the TapeStation DNA 1000 TapeScreen Assay (Agilent). Cluster generation was performed immediately before sequencing on a cBot with HiSeq^®^ PE Cluster Kit v4-cBot. The sequencing was conducted using a HiSeq^®^ SBS Kit on a HiSeq^®^ 2500, operating with HiSeq Control Software v2.2.68 and base-calling with Real Time Analysis (RTA) v1.18.66.3 (Illumina). Raw RNA-Seq short read data for all samples are freely available on NCBI under BioProject PRJNA482687.

### 4.3. De Novo Transcriptome Assembly and Quality Control

RNA-Seq reads for all 15 samples were corrected using the software Rcorrector (v1.0.2) [84]. Transcriptomes of all 15 samples were individually assembled from their RNA-Seq data, de novo. The assembly was carried out using Trinity (v2.4.0, Broad Institute of Massachusetts Institute of Technology and Harvard, Cambridge, MA, USA) [85,86]. The quality of the de novo transcriptome assembly was assessed using TransRate (v1.0.3, University of Cambridge, Cambridge, UK) [87] and BUSCO (Benchmarking Universal Single-Copy Orthologs) (v1.2, Swiss Institute of Bioinformatics, Geneva, Switzerland) [88] using the arthropoda odb9 database [89]. The quality score, also known as the TransRate score, is a score between 0.0–1.0 that is obtained by multiplying the mean of individual contig scores by the proportion of read pairs (original sequencing reads) that supported the transcriptome [87,90]. The results of BUSCO assessment are given in percentages of complete (C), fragmented (F) and missing (M) genes within the transcriptome [88]. Using *L. vannamei* as an example, stepwise methods of sample extraction, sequencing, de novo transcriptome assembly and quality check are summarised and schematically represented in Figure 1A.

### 4.4. Removal of the Inconclusive Dataset

Using the Rcorrected reads in an Assembly and Alignment-Free (AAF) method to create a phylogeny [91], it was discovered that one replicate of *M. latisulcatus* grouped with *M. endeavouri* rather than with the other two replicates of *M. latisulcatus*. To confirm the potentially misidentified sample, the assembled transcriptome was BLAST searched against the other *M. latisulcatus* and *M. endeavouri* transcriptomes, where the potentially misidentified sample also showed more similarity to *M. endeavouri*. Lastly, the transcriptomes were compared to known sequences of Enolase [92], which also confirmed that the misidentified sample is not *M. latisulcatus*.

### 4.5. Allergen Reference Database Construction

Known allergen AA sequences were retrieved from two reputable and peer-reviewed online databases to construct a reference allergen database for this study. The first is the World Health Organization & International Union of Immunological Societies (WHO/IUIS) Allergen Nomenclature database (www.allergen.org) [27]. The second is the AllergenOnline: The Food Allergy Research and Resource Program (FARRP) Allergen Protein database (v.17) (www.allergenonline.org) [28,29]. At the time of retrieval, the WHO/IUIS Allergen Nomenclature database contained 875 allergen AA sequences while the AllergenOnline database contained 2035 allergen AA sequences [27,28,29]. After removing duplicates between the 2 databases, a total of 2172 allergen AA sequences were compiled to form the reference allergen database for this study. 

### 4.6. BLAST Search for Allergens

The allergen database and the assembled transcripts for all 15 samples were imported into the Geneious™ software (v8.1.9, Biomatters, Auckland, New Zealand). In order to compare and search for transcripts that contain similar sequences to the allergen sequences compiled in the allergen database, blastx searches were carried out using the BLAST (Basic Local Alignment Search Tool) module within the Geneious™ software. The criteria for the search conducted are shown in Table S1.

The BLAST search results were filtered for matched sequences with a PI of 50% or more. Subject coverage (percentage of the allergen sequence that is covered by the matching transcript from the transcriptome) was manually calculated using the formula: Subject coverage = Sequence length/Subject length × 100%, where ‘sequence length’ is the length of the matched consensus sequence and the ‘subject length’ is the actual length of the allergen sequence from the constructed database. Results were then filtered again by selecting only sequences that have 90% or more subject coverage.

Duplicates of allergen sequences that aligned with contigs within the transcriptome were removed by keeping the top-matched allergen-transcript consensus sequence. The BLAST search results of 3 replicates of each species were then combined to form one list of allergens for every species and the duplicates (between replicates) were removed. Stepwise methods of allergen database construction and the processing of transcriptome data such as BLAST search, results refinement and removal of duplicates are schematically represented in Figure 1, using the three assembled transcriptome replicates of *L. vannamei* as an example. 

### 4.7. Analysing the BLAST Search Results

For each shrimp species, the matched allergen AA sequences were grouped into: ‘Shellfish’, ‘Mites’, ‘Insects’, ‘Fungi’, ‘Plants’, ‘Fish’ and ‘Other’, based on the organism that the allergen was documented in. The proportion of allergen sequences belonging to each group were graphed into a pie chart using GraphPad Prism (v8.4.3, GraphPad Software, San Diego, CA, USA) to show their distribution amongst different groups of allergen sources.

Multiple sequence alignment was conducted on all the contigs/transcripts that matched tropomyosin allergen in all five transcriptomes with shellfish tropomyosin allergens’ sequences (as reference). Mites’ and cockroaches’ tropomyosin allergen sequences were also included in the multiple sequence alignment that was conducted in Jalview2.1 using Clustal Omega [93]. Comparative AA sequence identities were carried out between the contigs from all five shrimp species that matched with tropomyosin, and previously reported crustacean, mites and cockroach tropomyosin allergens using Clustal Omega, EMBL-EBI. The multiple sequence alignment and comparative sequence identities were carried out for other documented crustacean allergens: Arginine kinase, myosin light chain, sarcoplasmic calcium-binding protein, troponin C, troponin I and triosephosphate isomerase. 

Non-crustacean allergens that have a PI value of more than 70% were shortlisted as highly likely candidates of unreported allergens in shrimp species. These unreported allergens were selected based on their match with the transcriptome of a minimum of 70% PI in at least one of the five shrimp species.

### 4.8. Measuring the Abundance of Allergen Sequences

Abundance of each transcript/contigs within the transcriptomes, in transcript-per-million (TPM) values, was quantified using Salmon software [94]. Briefly, Salmon is a software that estimates the abundance of each contig by measuring the number of reads from the RNA-Seq data that align to the contig being measured [94]. Abundance estimation values for all known crustacean allergens were retrieved from all 15 samples. For each allergen in each sample, the estimated abundance value is the sum of all TPM values of all the contigs that matched with that allergen. The mean TPM values with standard deviation error bars for each allergen of the three replicates for each shrimp species are graphically represented in Figure 7 and Figure 8. Standard deviation error bars were omitted from *M. latisulcatus* samples as only 2 replicates were investigated in this study. We first analysed the difference in abundance of all contigs representing a specific allergen, between the 5 shrimp species (Figure 7). In order to look for significant differences between two contigs representing the same allergen, we used unpaired *t*-test using GraphPad Prism (v8.4.3, GraphPad Software, San Diego, CA, USA). Next, we analysed the difference in abundance of allergens within each shrimp species (Figure 8). For these analyses, we only took into account the contig with the highest abundance, when there are more than one contig representing one allergen. To analyse significant differences between the seven crustacean allergens’ abundance, we used One-way ANOVA test using GraphPad Prism version 7.03 for Windows.

### 4.9. Molecular Phylogenetic Tree Building of TM, AK, MLC and SCP

Published AA sequences of the four widely studied crustacean allergens, TM, AK, MLC and SCP belonging to edible crustacean and mollusc species; and allergy-causing mite and insect species were extracted from NCBI Genbank and UniProt databases. The proteins that are not registered as an allergen in WHO/IUIS or AllergenOnline databases were also included. Molecular phylogenetic trees for each protein were built using MEGA X software (v10.0.5, Pennsylvania State University, State College, PA, USA) to determine the evolutionary distance between the same proteins from different species. The trees were constructed using the neighbour-joining method with the Poisson correction model. Hence, the branch lengths are the proportion of AA substitutions per site. Bootstrap test was also included (10,000 replicates), and the percentages are shown next to the branches. The gaps that occurred in alignment were treated as pairwise deletion.

## Figures and Tables

**Table 1:** Results of Trinity transcriptome assembly, TransRate, and BUSCO. Shrimp species name (common name) and their 1–3 biological replicates are shown with the transcriptomes’ number of contigs and assembly size after assembly by Trinity. TransRate and BUSCO scores (C: Complete, F: Fragmented, M: Missing) of each transcriptome are indicated.

Shrimp Species		Replicates	RNA-Seq	Transcriptome Assembly Metrics			Transrate Quality Assessment		BUSCO Scores		
			Normalised Read Count	No. of Contigs	Assembly Size	GC Content (%)	Proportion of Read Pairs Mapped (%)	Assembly Score	Complete (%)	Fragmented (%)	Missing (%)
** 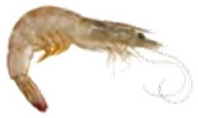 **	***L. vannamei*** (Whiteleg shrimp)	1	1,412,587	32,302	28.6 Mb	43.4	93.2	0.413	56	21	23
		2	1,412,010	33,574	29.4 Mb	43.0	92.6	0.401	56	23	21
		3	1,070,376	28,101	22.7 Mb	44.8	92.8	0.419	48	25	27
** 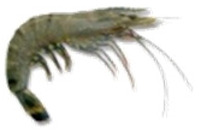 **	***P. monodon*** (Black Tiger shrimp)	1	1,609,374	41,971	37.9 Mb	44.3	91.9	0.387	66	20	14
		2	1,443,066	40,927	36.5 Mb	45.1	91.0	0.364	66	19	14
		3	1,643,259	42,510	38.1 Mb	43.7	92.3	0.390	64	21	14
** 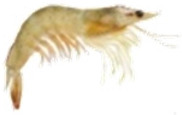 **	***F. merguiensis*** (Banana shrimp)	1	1,486,264	37,572	31.4 Mb	43.0	91.8	0.385	64	17	19
		2	1,657,940	41,336	34.8 Mb	42.6	91.7	0.385	67	16	17
		3	1,602,775	38,638	33.5 Mb	42.5	92.6	0.389	65	19	16
** 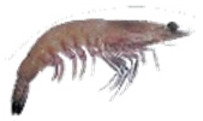 **	***M. latisulactus*** (King shrimp)	1	1,130,898	37,128	25.6 Mb	42.9	90.7	0.410	46	26	27
		2	1,052,237	28,125	21.7 Mb	42.8	92.2	0.411	43	25	32
** 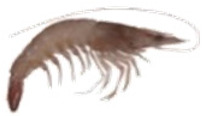 **	***M. endeavouri*** (Endeavour shrimp)	1	1,142,169	35,407	25.9 Mb	42.5	90.6	0.374	48	25	27
		2	1,035,324	30,879	23.2 Mb	42.3	91.2	0.399	48	24	27
		3	1,081,301	38,204	25.5 Mb	43.3	87.9	0.355	49	26	25

**Table 2:** List of unreported potential allergens identified that have a minimum of 70% pairwise identity value in at least one species. List includes protein name, the common and scientific name of the allergen source, along with the allergen’s IUIS nomenclature, % Pairwise identity, and E-values. % Pairwise identity values are in bold and E-values are in brackets. LV: *L. vannamei*, PM: *P. monodon*, FM: *F. merguiensis*, ML: *M. latisulcatus*, ME: *M. endeavouri*.

Allergens				LV Whiteleg Shrimp	PM Black Tiger Shrimp	FM Banana Shrimp	ML King Shrimp	ME Endeavour Shrimp
Protein Name	Source Name		IUIS Nomenclature					
	Common	Scientific						
Heat Shock Protein 70	Storage mite	*Tyrophagus putrescentiae*	Tyr p 28	**85.1%** (0)	**82.7%** (0)	**83.3%** (0)	**82.7%** (0)	**84.3%** (0)
Alpha-Tubulin	American house dust mite	*Dermatophagoides farinae*	Der f 33	**81.8**% (0)	**81.7%** (0)	**81.6%** (0)	**81.6%** (0)	**81.6%** (0)
Chymotrypsin	American house dust mite	*Dermatophagoides farinae*	Der f 6	**78.7%** (4.3 × 10^−94^)	**78.7%** (2.13 × 10^−94^)	**79.3%** (1.45 × 10^−94^)	**79.9%** (3.71 × 10^−97^)	**80.5%** (3.93 × 10^−95^)
Enolase 3–2	Atlantic salmon	*Salmo salar*	Sal s 2	**74.8%** (0)	**74.6%** (0)	**74.1%** (0)	**74.6%** (0)	**74.5%** (0)
Aldolase A	Yellowfin tuna	*Thunnus albacares*	Thu a 3	**66.0%** (2.29 × 10^−164^)	**64.9%** (2.25 × 10^−164^)	**70.1%** (4.09 × 10^−169^)	**69.6%** (1.47 × 10^−166^)	**70.1%** (3.65 × 10^−167^)

## Data Availability

Raw RNA-Seq short read data for all samples are freely available on NCBI under BioPro-ject PRJNA482687 at https://www.ncbi.nlm.nih.gov/bioproject/482687.

## Supplementary Materials

The following are available online at https://www.mdpi.com/1422-0067/22/1/32/s1. Figure S1. Multiple sequence alignment of tropomyosin (TM) allergen. Sequences consist of (1–2) known shrimp TM allergen, (3–9) contigs from five shrimp species that matched with TM allergen and (10–12) TM allergen sequences from house dust mite and cockroaches. Multiple sequence alignment was conducted in Jalview 2.1 using Clustal Omega. Figure S2. Multiple sequence alignment of arginine kinase (AK) allergen. Sequences consist of (1–2) known shrimp AK allergen, (3–8) contigs from five shrimp species that matched AK allergen and (9–12) AK allergen sequences from house dust mites and cockroaches. Multiple sequence alignment was conducted in Jalview 2.1 using Clustal Omega. Figure S3. Multiple sequence alignment of myosin light chain (MLC) allergen. Sequences consist of (1–3) known shrimp MLC allergen, (4–8) contigs from five shrimp species that matched with MLC allergen and (9–10) house dust mite and cockroach MLC allergen. Multiple sequence alignment was conducted in Jalview 2.1 using Clustal Omega. Figure S4. Multiple sequence alignment of sarcoplasmic calcium-binding protein (SCP) allergen. Sequences consist of (1–3) known shrimp SCP allergen and (4–12) contigs from five shrimp species that matched with SCP allergen. Multiple sequence alignment was conducted in Jalview 2.1 using Clustal Omega. Figure S5. Comparison of troponin C (TNC) allergen. Comparison of amino acid sequence identities of (1–7) contigs from five shrimp species that matched with TNC allergen, (8–9) known shrimp TNC allergen, and (10–14) cockroach and storage mite TNC allergen. The sequence identities were calculated using multiple sequence alignment in Clustal Omega (EMBL-EBI). Figure S6. Multiple sequence alignment of troponin C (TNC) allergen. Sequences consist of (1–2) known shrimp TNC allergen, (3–9) contigs from five shrimp species that matched with TNC allergen and (10–14) TNC allergen sequences from house dust mites and cockroaches. Multiple sequence alignment was conducted in Jalview 2.1 using Clustal Omega. Figure S7. Comparison of troponin I (TNI) allergen. Comparison of amino acid sequence identities of (1–5) contigs from five shrimp species that matched with TNI allergen and (6) known crayfish TNI allergen. The sequence identities were calculated using multiple sequence alignment in Clustal Omega (EMBL-EBI). Figure S8. Multiple sequence alignment of troponin I (TNI) allergen. Sequences consist of (1) known crayfish TNI allergen and (2–6) contigs from five shrimp species that matched with TNI allergen. Multiple sequence alignment was conducted in Jalview 2.1 using Clustal Omega. Figure S9. Comparison of triosephosphate isomerase (TIM) allergen. Comparison of amino acid sequence identities of (1–5) contigs from five shrimp species that matched with TIM allergen, (6) known shrimp TIM allergen, and (7–8) house dust mite TIM allergen. The sequence identities were calculated using multiple sequence alignment in Clustal Omega (EMBL-EBI). Figure S10. Multiple sequence alignment of triosephosphate isomerase (TIM) allergen. Sequences consist of (1) known shrimp TIM allergen, (2–6) contigs from five shrimp species that matched with TIM allergen and (7–8) TIM allergen sequences from house dust mites. Multiple sequence alignment was conducted in Jalview 2.1 using Clustal Omega. Table S1. Criteria used in the BLAST search. The criteria shown here are only for the BLAST search utility within the Geneious™ software. Additional search criteria (for this project) were later used in the refining process of the search results, e.g., Minimum % Pairwise Identity of 50%.
