# Supplementary material for: Novel Allergen Discovery through Comprehensive De Novo Transcriptomic Analyses of Five Shrimp Species
Source: Int J Mol Sci. 2020 Dec 22;22(1):32. doi: 10.3390/ijms22010032 (PMC7792927; doi:10.3390/ijms22010032)
Supplement: Supplementary file 1 [file ijms-22-00032-s001.zip › Figure1.pptx]

## Slide 1
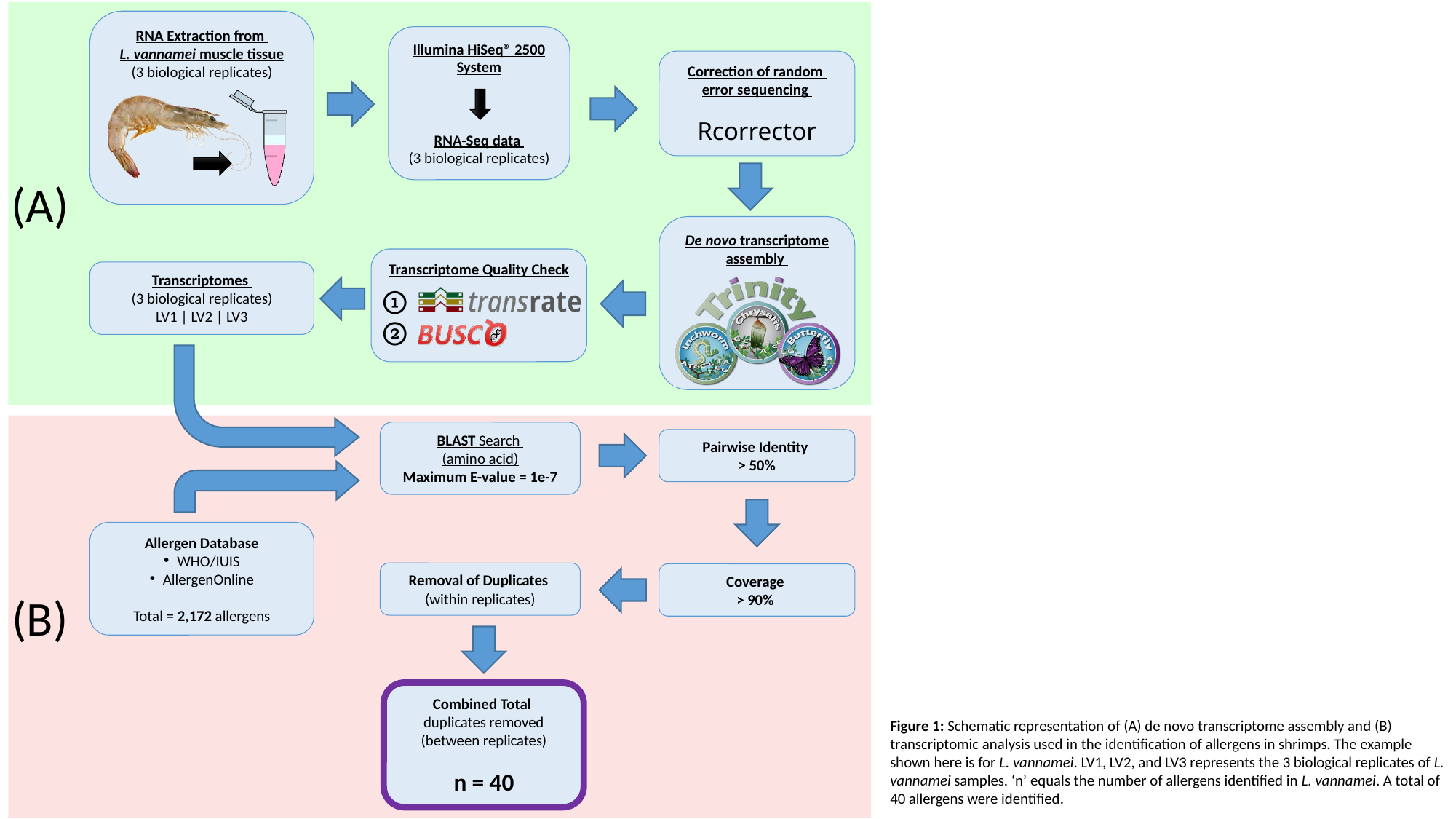

RNA Extraction from
L. vannamei muscle tissue
(3 biological replicates)
Illumina HiSeq® 2500 System
RNA-Seq data
(3 biological replicates)
Correction of random
error sequencing
Rcorrector
(A)
De novo transcriptome assembly
Transcriptome Quality Check
Transcriptomes
(3 biological replicates)
LV1 | LV2 | LV3
BLAST Search
(amino acid)
Maximum E-value = 1e-7
Pairwise Identity
> 50%
Allergen Database
WHO/IUIS
AllergenOnline
Total = 2,172 allergens
Removal of Duplicates
(within replicates)
Coverage
> 90%
(B)
Combined Total
duplicates removed (between replicates)
n = 40
Figure 1: Schematic representation of (A) de novo transcriptome assembly and (B) transcriptomic analysis used in the identification of allergens in shrimps. The example shown here is for L. vannamei. LV1, LV2, and LV3 represents the 3 biological replicates of L. vannamei samples. ‘n’ equals the number of allergens identified in L. vannamei. A total of 40 allergens were identified.
