# Supplementary material for: Novel Allergen Discovery through Comprehensive De Novo Transcriptomic Analyses of Five Shrimp Species
Source: Int J Mol Sci. 2020 Dec 22;22(1):32. doi: 10.3390/ijms22010032 (PMC7792927; doi:10.3390/ijms22010032)
Supplement: Supplementary file 1 [file ijms-22-00032-s001.zip › Figure2.pptx]

## Slide 1
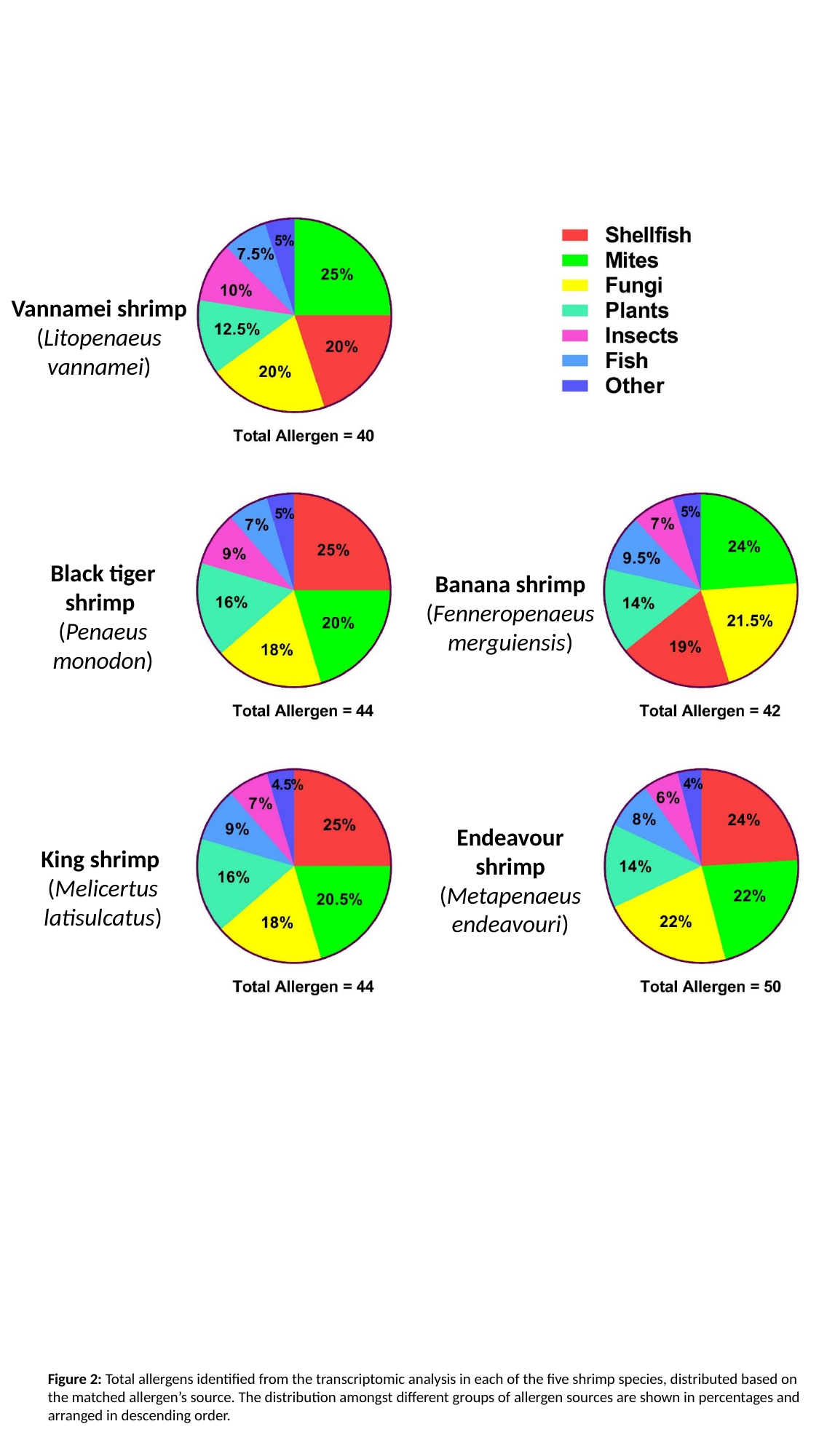

Vannamei shrimp (Litopenaeus vannamei)
Black tiger shrimp
(Penaeus monodon)
Banana shrimp (Fenneropenaeus merguiensis)
Endeavour shrimp (Metapenaeus endeavouri)
King shrimp
(Melicertus latisulcatus)
Figure 2: Total allergens identified from the transcriptomic analysis in each of the five shrimp species, distributed based on the matched allergen’s source. The distribution amongst different groups of allergen sources are shown in percentages and arranged in descending order.
