# Supplementary material for: Novel Allergen Discovery through Comprehensive De Novo Transcriptomic Analyses of Five Shrimp Species
Source: Int J Mol Sci. 2020 Dec 22;22(1):32. doi: 10.3390/ijms22010032 (PMC7792927; doi:10.3390/ijms22010032)
Supplement: Supplementary file 1 [file ijms-22-00032-s001.zip › Figure5.pptx]

## Slide 1
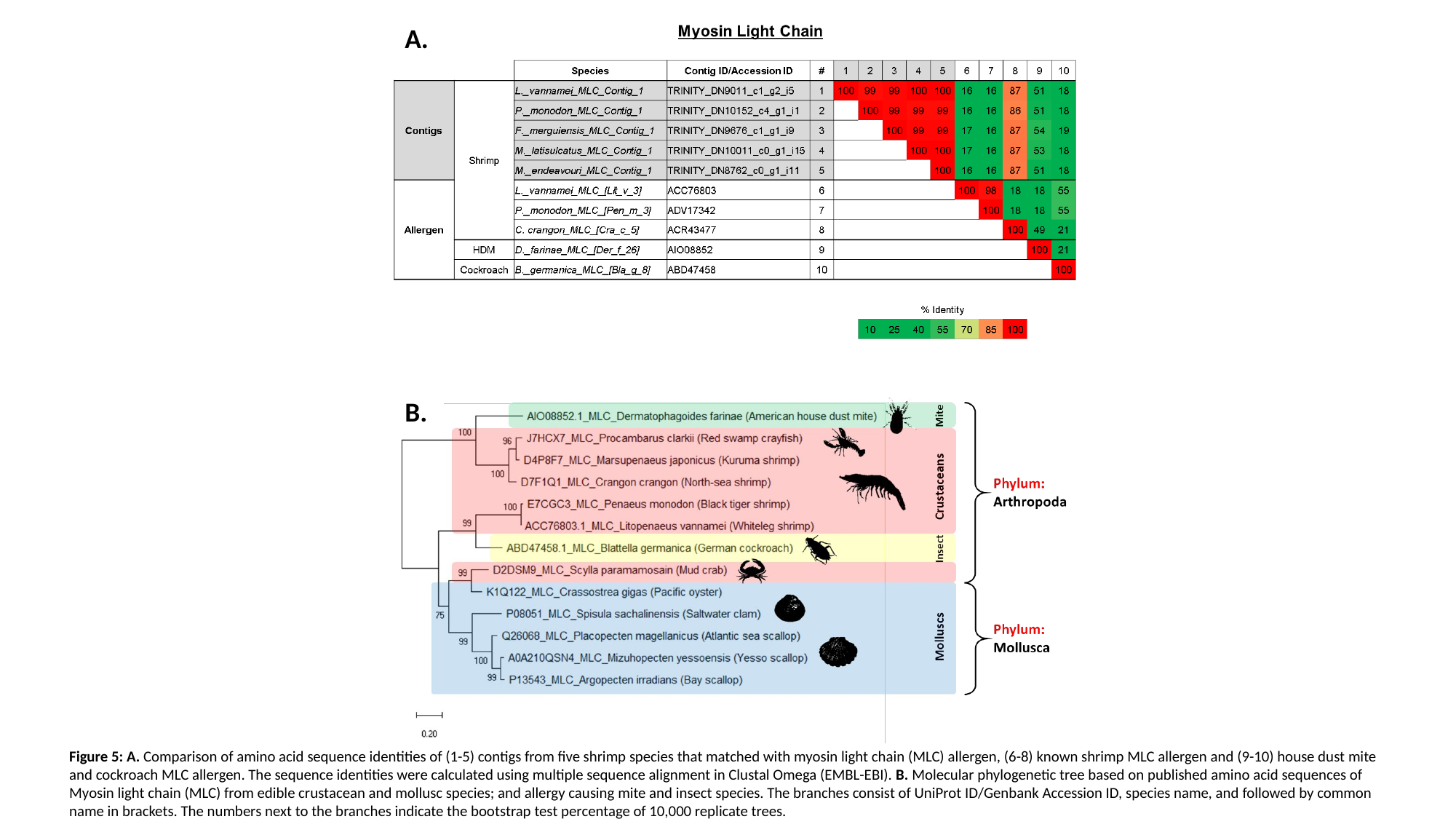

Figure 5: A. Comparison of amino acid sequence identities of (1-5) contigs from five shrimp species that matched with myosin light chain (MLC) allergen, (6-8) known shrimp MLC allergen and (9-10) house dust mite and cockroach MLC allergen. The sequence identities were calculated using multiple sequence alignment in Clustal Omega (EMBL-EBI). B. Molecular phylogenetic tree based on published amino acid sequences of Myosin light chain (MLC) from edible crustacean and mollusc species; and allergy causing mite and insect species. The branches consist of UniProt ID/Genbank Accession ID, species name, and followed by common name in brackets. The numbers next to the branches indicate the bootstrap test percentage of 10,000 replicate trees.
