# Supplementary material for: Novel Allergen Discovery through Comprehensive De Novo Transcriptomic Analyses of Five Shrimp Species
Source: Int J Mol Sci. 2020 Dec 22;22(1):32. doi: 10.3390/ijms22010032 (PMC7792927; doi:10.3390/ijms22010032)
Supplement: Supplementary file 1 [file ijms-22-00032-s001.zip › Figure7.pptx]

## Slide 1
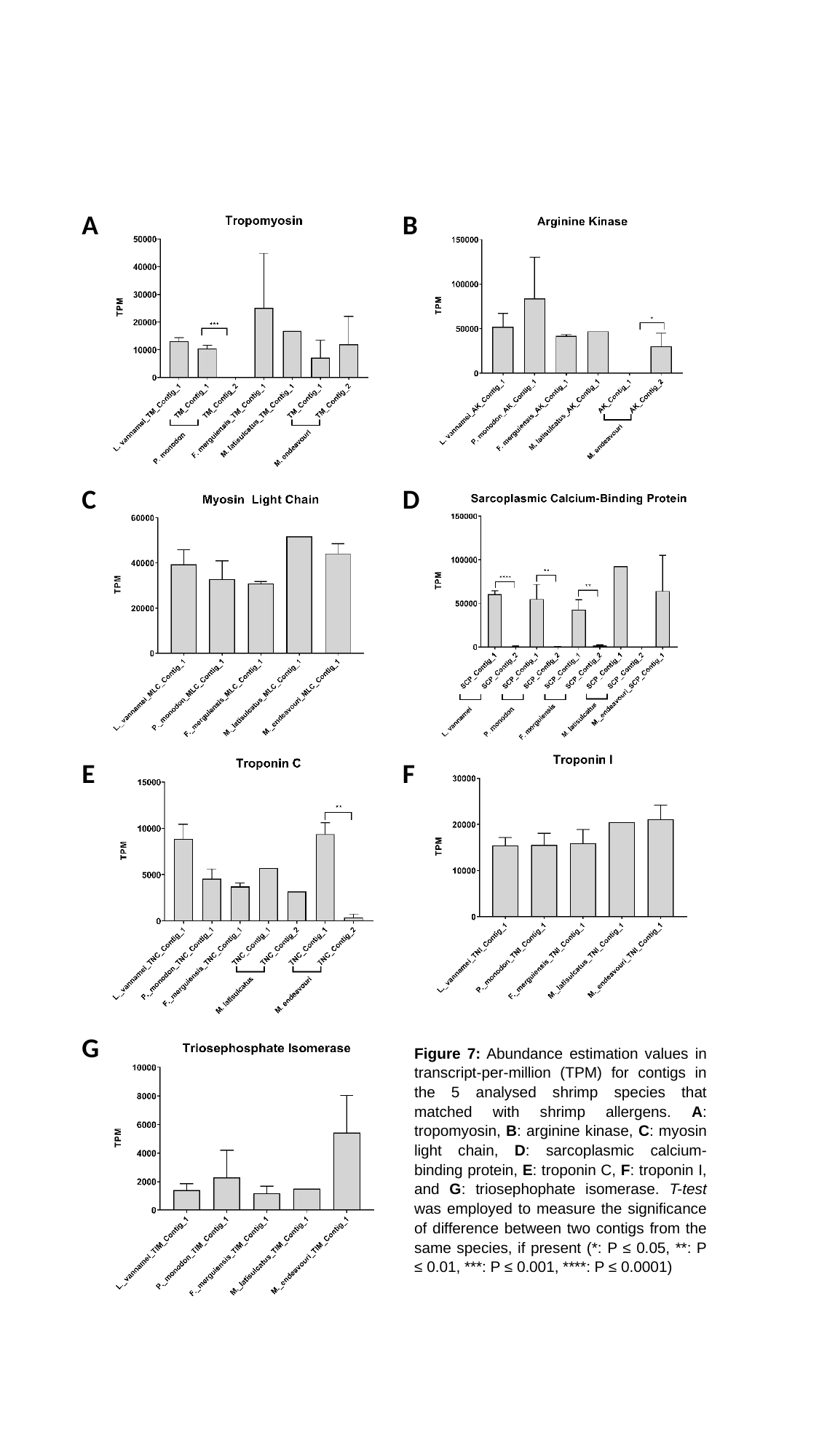

A
B
C
D
E
F
G
Figure 7: Abundance estimation values in transcript-per-million (TPM) for contigs in the 5 analysed shrimp species that matched with shrimp allergens. A: tropomyosin, B: arginine kinase, C: myosin light chain, D: sarcoplasmic calcium-binding protein, E: troponin C, F: troponin I, and G: triosephophate isomerase. T-test was employed to measure the significance of difference between two contigs from the same species, if present (*: P ≤ 0.05, **: P ≤ 0.01, ***: P ≤ 0.001, ****: P ≤ 0.0001)
