# Supplementary material for: Novel Allergen Discovery through Comprehensive De Novo Transcriptomic Analyses of Five Shrimp Species
Source: Int J Mol Sci. 2020 Dec 22;22(1):32. doi: 10.3390/ijms22010032 (PMC7792927; doi:10.3390/ijms22010032)
Supplement: Supplementary file 1 [file ijms-22-00032-s001.zip › Figure8.pptx]

## Slide 1
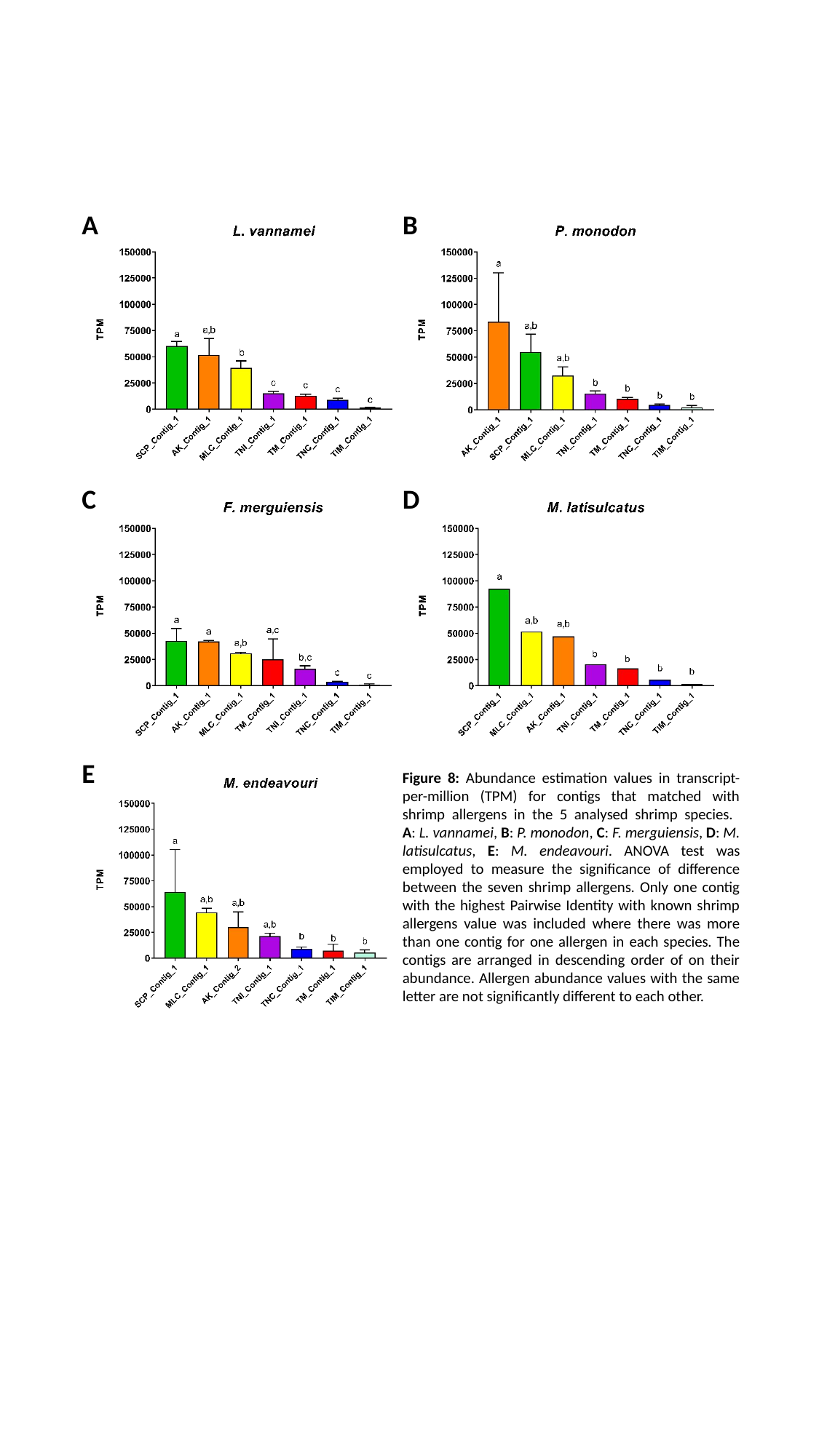

A
B
C
D
E
Figure 8: Abundance estimation values in transcript-per-million (TPM) for contigs that matched with shrimp allergens in the 5 analysed shrimp species.
A: L. vannamei, B: P. monodon, C: F. merguiensis, D: M. latisulcatus, E: M. endeavouri. ANOVA test was employed to measure the significance of difference between the seven shrimp allergens. Only one contig with the highest Pairwise Identity with known shrimp allergens value was included where there was more than one contig for one allergen in each species. The contigs are arranged in descending order of on their abundance. Allergen abundance values with the same letter are not significantly different to each other.
