# Supplementary material for: Novel Allergen Discovery through Comprehensive De Novo Transcriptomic Analyses of Five Shrimp Species
Source: Int J Mol Sci. 2020 Dec 22;22(1):32. doi: 10.3390/ijms22010032 (PMC7792927; doi:10.3390/ijms22010032)
Supplement: Supplementary file 1 [file ijms-22-00032-s001.zip › Table1.docx]

**Table 1 Results of Trinity transcriptome assembly, TransRate, and BUSCO**

| Shrimp species | | Replicates | RNA-Seq | Transcriptome assembly metrics | | | Transrate quality assessment | | BUSCO scores | | |
| --- | --- | --- | --- | --- | --- | --- | --- | --- | --- | --- | --- |
|  |  |  | **Normalized read count** | **No. of contigs** | **Assembly size** | **GC content (%)** | **Proportion of read pairs mapped (%)** | **Assembly score** | **Complete (%)** | **Fragmented (%)** | **Missing (%)** |
| 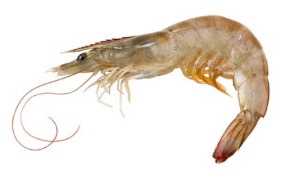 | ***L. vannamei***  (Whiteleg shrimp) | 1 | 1,412,587 | 32,302 | 28.6Mb | 43.4 | 93.2 | 0.413 | 56 | 21 | 23 |
|  |  | 2 | 1,412,010 | 33,574 | 29.4Mb | 43.0 | 92.6 | 0.401 | 56 | 23 | 21 |
|  |  | 3 | 1,070,376 | 28,101 | 22.7Mb | 44.8 | 92.8 | 0.419 | 48 | 25 | 27 |
| 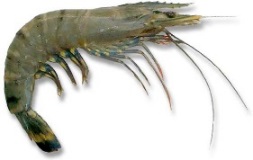 | ***P. monodon***  (Black Tiger shrimp) | 1 | 1,609,374 | 41,971 | 37.9Mb | 44.3 | 91.9 | 0.387 | 66 | 20 | 14 |
|  |  | 2 | 1,443,066 | 40,927 | 36.5Mb | 45.1 | 91.0 | 0.364 | 66 | 19 | 14 |
|  |  | 3 | 1,643,259 | 42,510 | 38.1Mb | 43.7 | 92.3 | 0.390 | 64 | 21 | 14 |
| 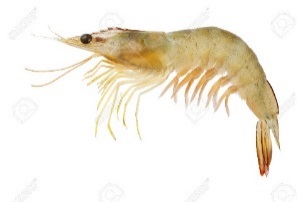 | ***F. merguiensis***  (Banana shrimp) | 1 | 1,486,264 | 37,572 | 31.4Mb | 43.0 | 91.8 | 0.385 | 64 | 17 | 19 |
|  |  | 2 | 1,657,940 | 41,336 | 34.8Mb | 42.6 | 91.7 | 0.385 | 67 | 16 | 17 |
|  |  | 3 | 1,602,775 | 38,638 | 33.5Mb | 42.5 | 92.6 | 0.389 | 65 | 19 | 16 |
| 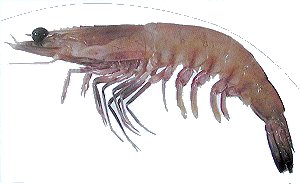 | ***M. latisulactus***  (King shrimp) | 1 | 1,130,898 | 37,128 | 25.6Mb | 42.9 | 90.7 | 0.410 | 46 | 26 | 27 |
|  |  | 2 | 1,052,237 | 28,125 | 21.7Mb | 42.8 | 92.2 | 0.411 | 43 | 25 | 32 |
| 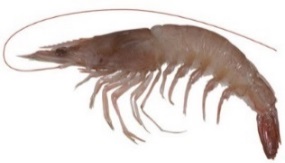 | ***M. endeavouri***  (Endeavour shrimp) | 1 | 1,142,169 | 35,407 | 25.9Mb | 42.5 | 90.6 | 0.374 | 48 | 25 | 27 |
|  |  | 2 | 1,035,324 | 30,879 | 23.2Mb | 42.3 | 91.2 | 0.399 | 48 | 24 | 27 |
|  |  | 3 | 1,081,301 | 38,204 | 25.5Mb | 43.3 | 87.9 | 0.355 | 49 | 26 | 25 |

*Note*: Shrimp species name (common name) and their 1-3 biological replicates are shown here with their transcriptomes’ number of contigs and assembly size after assembly by Trinity. TransRate score and BUSCO scores (C: complete, F: fragmented, M: missing) of each transcriptome are also shown here.
