# Supplementary material for: Novel Allergen Discovery through Comprehensive De Novo Transcriptomic Analyses of Five Shrimp Species
Source: Int J Mol Sci. 2020 Dec 22;22(1):32. doi: 10.3390/ijms22010032 (PMC7792927; doi:10.3390/ijms22010032)
Supplement: Supplementary file 1 [file ijms-22-00032-s001.zip › Table2.docx]

**Table 2 List of unreported allergens identified that have a minimum of 70% pairwise identity value in at least one species**

| **Allergens** | | | | **LV Whiteleg shrimp**  (E Value) | **PM Black tiger shrimp**  (E Value) | **FM Banana shrimp**  (E Value) | **ML King  shrimp**  (E Value) | **ME Endeavour shrimp**  (E Value) |
| --- | --- | --- | --- | --- | --- | --- | --- | --- |
| **Protein name** | **Source name** | | **IUIS nomen-clature** |  |  |  |  |  |
|  | **Common** | **Scientific** |  |  |  |  |  |  |
| **Heat shock protein 70** | Storage mite | *Tyrophagus putrescentiae* | Tyr p 28 | **85.1%**  (0) | **82.7%**  (0) | **83.3%**  (0) | **82.7%**  (0) | **84.3%**  (0) |
| **Alpha-tubulin** | American house dust mite | *Dermatophagoides farinae* | Der f 33 | **81.8%**  (0) | **81.7%**  (0) | **81.6%**  (0) | **81.6%**  (0) | **81.6%**  (0) |
| **Chymotrypsin** | American house dust mite | *Dermatophagoides farinae* | Der f 6 | **78.7%**  (4.3E-94) | **78.7%**  (2.13E-94) | **79.3%**  (1.45E-94) | **79.9%**  (3.71E-97) | **80.5%**  (3.93E-95) |
| **Enolase 3-2** | Atlantic salmon | *Salmo salar* | Sal s 2 | **74.8%**  (0) | **74.6%**  (0) | **74.1%**  (0) | **74.6%**  (0) | **74.5%**  (0) |
| **Glyceral-dehyde-3-phosphate de-hydrogenase** | Wheat | *Triticum aestivum* | Tri a 34 | **72.3%**  (1.25E-168) | **72.0%**  (2.87E-172) | **71.7%**  (1.86E-170) | **72.0%**  (1.21E-174) | **72.3%**  (4.91E-175) |
| **Cyclophilin** | Common mould | *Aspergillus fumigatus* | Asp f 27 | **61.9%**  (8.45E-65) | **62.5%**  (1.87E-67) | **70.3%**  (2.63E-75) | **70.7%**  (4.38E-75) | **69.3%**  (5.53E-76) |
| **Aldolase A** | Yellowfin tuna | *Thunnus albacares* | Thu a 3 | **66.0%**  (2.29E-164) | **64.9%**  (2.25E-164) | **70.1%**  (4.09E-169) | **69.6%**  (1.47E-166) | **70.1%**  (3.65E-167) |

*Note*: List includes protein name, the common and scientific name of the allergen source, along with the allergen sequence’s IUIS nomenclature. % Pairwise identity and E-values. LV: *L. vannamei*, PM: *P. monodon*, FM: *F. merguiensis*, ML: *M. latisulcatus*, ME: *M. endeavouri*. Proteins with a % Pairwise identity of 70% or higher (highly likely to be allergenic) are highlighted in red.
